# Neutrophil-to-Lymphocyte Ratio, Platelet-to-Lymphocyte Ratio, and Their Variations as a Basis for a Prediction Model in Advanced NSCLC Patients Receiving Anlotinib

**DOI:** 10.1155/2022/5879137

**Published:** 2022-03-20

**Authors:** Tian Chen, Chao Song, Gaofeng Liang, Xiaoyu Xu, Chen Wang, Zhanchun Zhang, Mengqiu Tang

**Affiliations:** ^1^Department of Radiation Oncology, Ningbo Medical Center Lihuili Hospital, Ningbo University, Ningbo, China; ^2^Department of Surgery, Yuyao Maternity and Child Health Care Hospital, Ningbo, China; ^3^Department of Thoracic Surgery, Ningbo Medical Center Lihuili Hospital, Ningbo University, Ningbo, China; ^4^Department of Gastroenterology, Ningbo Medical Center Lihuili Hospital, Ningbo University, Ningbo, China

## Abstract

**Background:**

A phase III randomized multicenter trial (ALTER0303) reported anlotinib to be significantly beneficial to patient survival. An array of inflammatory biomarkers, such as neutrophil lymphocyte ratio (NLR) and platelet lymphocyte ratio (PLR), are associated with the response to treatment in numerous types of cancer. However, we found few studies investigating the predictive value of NLR or PLR in advanced NSCLC treatment with anlotinib. Thus, our objective was to examine the relationship between NLR and PLR and treatment effect, as well as to individuate patient stratification and selection.

**Methods:**

NLR and PLR as well as their variations were calculated in 152 advanced NSCLC patients receiving anlotinib as a third or further-line treatment at Ningbo Medical Center Lihuili Hospital between July 2018 and December 2020. The best cut-off values of NLR and PLR for predicting the treatment response were selected. Survival curves were plotted using the Kaplan–Meier method, while univariable and multivariable Cox regression were used to identify and determine dependent and independent predictors of survival.

**Results:**

, Low disease control rate (DCR) was related with a high pre-NLR (*P* = 0.007), high pre-PLR (*P* = 0.004), and elevated post-NLR (*P* = 0.010). Multivariate analysis determined high pre-PLR (>205.63) and elevated post-NLR to be independently associated with poor PFS or OS. Patients whose risk score was 2 resulting from the prediction model based on pre-PLR and post-NLR had a 4.52 times higher risk of death compared to patients whose risk score was 0 (HR: 4.516, 95% CI: 2.502-8.152, *P* ≤ 0.001).

**Conclusions:**

Pre-PLR and post-NLR were independent prognostic indicators in patients with advanced NSCLC receiving anlotinib as a third or further-line treatment. Patients whose risk value score was 0 had a higher therapy effectiveness and better survival.

## 1. Background

Lung cancer is one of the most common malignancies and a leading cause of cancer-related death worldwide.

Non-small cell lung cancer (NSCLC) accounts for 85% of all lung cancers, a majority of which present with advanced metastatic disease [1, 2]. The National Comprehensive Cancer Network (NCCN) guidelines provided guidance for only first- and second-line regimens [3]. Anlotinib has been approved as a third-line therapy for advanced NSCLC according to the guidelines by the Chinese Society of Clinical Oncology (CSCO). Anlotinib is a new multitargeting tyrosine kinase inhibitor (TKI) that acts on tumor angiogenesis and proliferation signaling. Its underlying targets include the vascular endothelium growth factor receptors 1 to 3, epidermal growth factor receptor (EGFR), the fibroblast growth factor receptors 1 to 4, the immune-derived growth factor receptor, and the stem cell factor receptor [4]. A randomized multicenter phase II trial (ALTER0302) reported that the objective response rate (ORR) for anlotinib was only 10.0% [5]. Similarly, anlotinib was associated with a 4-month improvement in the median progress free survival (mPFS) and a 3.3-month improvement in the median overall survival compared to the control in the same study. For patients in whom disease control rate (DCR) was achieved, the shortest response duration was 1.5 months and the longest at least 18 months [6]. It is critically important to effectively screen out patients who may benefit most from anlotinib therapy by identifying viable and good predictors. Recently, some studies have increasingly identified a couple of factors that may potentially predict the treatment effectiveness of anlotinib. Wang et al. retrospectively reviewed and analyzed the prognostic factors of the ALTER0303 trial. They concluded that common adverse effects of anlotinib therapy had an intimate connection to patient prognosis and survival [7]. It is generally understood that individualized therapy is predicated on the identification and standardization of pretreatment biomarkers to predict treatment prognosis and outcome. However, studies focused on the potential predictive markers before treatment are scanty to say the least.

Previous studies have demonstrated that the systemic infection response plays a critical role in the symptom response [8, 9]. Many clinical inflammation markers, such as neutrophil lymphocyte ratio (NLR) and platelet lymphocyte ratio (PLR), can be easily measured via relatively inexpensive tests and with accurately reliable clinical results in several types of cancers and other diseases [10–17]. There have also been articles reporting that the predicting value of NLR and PLR in not only viable foe surgical treatment of early stage NSCLC but also in chemo-radiotherapy and immunotherapy of advanced NSCLC [13, 18–20]. Furthermore, researchers have considered them useful biomarkers to predict the therapeutic effect of several antiangiogenic drugs (such as regorafenib, sorafenib, and renvastinib) in advanced colorectal and liver cancers [21–24]. However, at present, we have found limited reports about the predicting value of NLR and PLR in advanced NSCLC patients treated with antiangiogenic therapy.

In this study, we retrospectively analyzed the prognostic and predictive role of NLR, PLR, and their variations in advanced NSCLC patients receiving anlotinib as a third or further-line treatment. The aim is to stratify and select individual, potentially reliable and convenient markers for patients.

## 2. Methods

### 2.1. Patient Selection

We retrospectively reviewed data of patients with advanced NSCLC receiving anlotinib as a third or further-line treatment at Ningbo Medical Center Lihuili Hospital between July 2018 and December 2020. The inclusion criteria were as follows: (1) a pathology diagnosis of stage IV NSCLC (recurrent or metastatic); (2) receiving at least two standard systemic therapeutic regimens; (3) treatment with anlotinib as a monotherapy for more than 2 weeks; (4) no obvious symptoms of infection or recent treatment with steroids; (5) no history of liver cirrhosis; and (6) available NLR and PLR data starting from 2 weeks before treatment to 2-4 weeks after treatment. A total of 152 patients were enrolled.

### 2.2. Data Collection and Definitions

Clinical characteristics and pathological findings, such as age, performance status, pathology, and history of tumor surgery, were recorded. Hematology data collected from 2 weeks prior to treatment to 2-4 weeks after treatment including neutrophil count (×109/L), platelet count (×109/L), and lymphocyte count (×109/L). We define the NLR as a neutrophil count divided by lymphocyte count, and PLR as a platelet count divided by lymphocyte count. Pretreatment NLR (pre-NLR) was determined within 2 weeks before treatment, while posttreatment NLR (post-NLR) within 2-4 weeks after treatment. Similarly, pretreatment PLR (pre-PLR) was recorded within 2 weeks before treatment and posttreatment PLR (post-PLR) within 2-4 weeks post treatment.

### 2.3. Anlotinib Treatment

Anlotinib was indicated for advanced NSCLC patients as a third or further-line treatment with an initial oral dose of 10-12 mg/day. Each cycle was defined as 2 weeks on-treatment followed by 1 week off-treatment. In case of an intolerable treatment-related adverse event, the dose would be cut back to 8-10 mg per day. Treatment was continued until disease progression or treatment intolerance.

### 2.4. Assessments of Therapeutic Response and Follow-Up

Follow-up evaluations, including B-ultrasound and computed tomography scan, were performed at 3 or 6 weeks after introducing anlotinib based on the Response Evaluation Criteria in Solid Tumors (RECIST) criteria. Systemic review was performed per every 2 cycles. Diagnostic examinations were performed when recurrence was suspected. DCR was defined as the percentage of patients evaluated who achieved complete response (CR), partial response (PR), and stable disease (SD). ORR was defined as the percentage of patients evaluated who achieved CR and PR. Progression free survival (PFS) was defined as the time from the onset of therapy and to progression or last time of follow up. The overall survival (OS) was calculated from the start of treatment to the time of death (irrespective of cause of death) or to last follow-up date.

### 2.5. Statistical Analysis

The statistical analysis was performed with SPSS (version 26.0, IBM) and R version 4.0.1 (R Foundation for Statistical Computing, Vienna, Austria). The best cut-off values of NLR and PLR from the maximum Youden's index, as well as the corresponding sensitivity and specificity for discriminating DCR and progressive disease (PD), were obtained from receiver operating characteristic (ROC) curve analysis. Using Fisher's exact or chi-square test, Kaplan–Meier method to plot survival curves and determine any differences by the univariable and multivariable Cox regressions. Variables with P value of <0.05 in the univariable analysis were considered for inclusion in the multivariable logistic regression model. The model was validated by the 5-fold cross-validation. Threshold for statistical significance was set at P < 0.05.

## 3. Results

### 3.1. Patient and Treatment Characteristics

A total of 152 patients who met the inclusion criteria were chosen to be included in our study. The basic patient characteristics are summarized in Table 1. The major driver alterations was EGFR mutation. Alk fusion mutation was noted in 1 case and of c-met springing in another. Of the 152 patients, 32 (21.1%) harbored EGFR mutations. 42 (27.6%) patients had a ECOG score greater than 2. Adenocarcinoma or squamous cell carcinoma aside, 2 cases were pathologically diagnosed as adenosquamous in nature and 1 as sarcomatoid. Patients with confirmed presence of mutation had prior treatment with targeted therapy. Patients initially treated with 10 mg/day of anlotinib accounted for 31(20.4%) of all patients.

With a median follow-up time of 7.05 months (1.1 to 24.4 months), 121 patients (79.6%) had died and all the patients were recrudescent at the end of the follow-up time. 10 (6.6%), 98 (64.5%), and 44 (28.9%) patients achieved PR, SD, and PD, respectively. None of them achieved a CR. DCR was achieved in 71.1% and ORR in 6.6% of patients. The median PFS and OS for the whole cohort of patients were 3.0 and 7.4 months, respectively.

### 3.2. Optimal Cut-off Values for NLR and PLR

**Figure 1:**
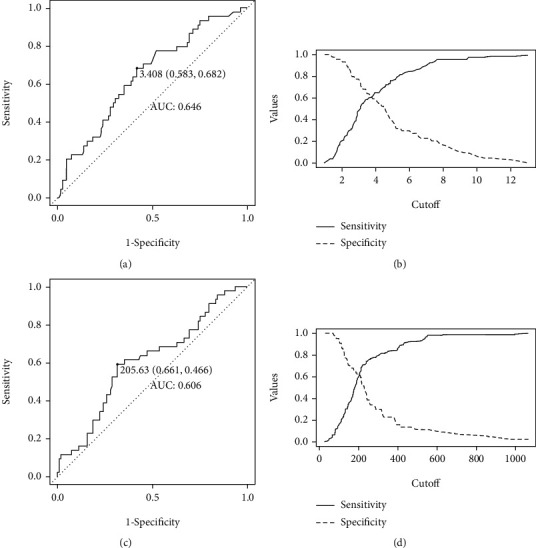
Receiver operating curves for treatment response were plotted to determine the optimum cut-off for pre-NLR (a, b) and pre-PLR (c, d).

Optimal thresholds for NLR and PLR for all patients in the study were determined using ROC curves. For pre-NLR, the optimal cut-off value was 3.41 and the area under the curve (AUC) was 0.646 (95% CI: 0.551 – 0.741), and sensitivity and specificity were 0.682 and 0.417, respectively (Figures 1(a) and 1(b)). On the other hand, the optimal cut-off value for pre-PLR was 205.63 with the concentration of 0.606 (95 percent CI: 0.506–0.705), sensitivity of 0.6614, and specificity of 0.4661 (Figures 1(c) and 1(d)).

### 3.3. Correlation between NLR, PLR, and Their Variations with Clinicopathological Parameters and Treatment Response

Before treatment, 74 (48.7%) patients had a high NLR > 3.41 and 78 (51.3%) had a low NLR ≤ 3.41. In addition, 66 (43.4%) had a high PLR > 205.63, while 86 (56.6%) had a low PLR ≤ 205.63. After treatment, 72 (47.4%) patients experienced an elevation in NLR and 80 (52.6%) a drop in NLR. PLR increased in 57 (37.5%) patients and the reverse occurred in 95 (62.5%) patients. A low DCR was associated with a high pre-NLR (*P* = 0.007), high pre-PLR (*P* = 0.004), and post-NLR elevation (*P* = 0.010), yet negatively related to a rise in post-PLR (*P* = 0.579). No significant correlation between ORR and any of the indicators was observed. Findings from the analysis pre-NLR and pre-PLR in relation to treatment response are detailed in Tables 2 and 3.

### 3.4. Factors Associated with Prognosis

**Figure 2:**
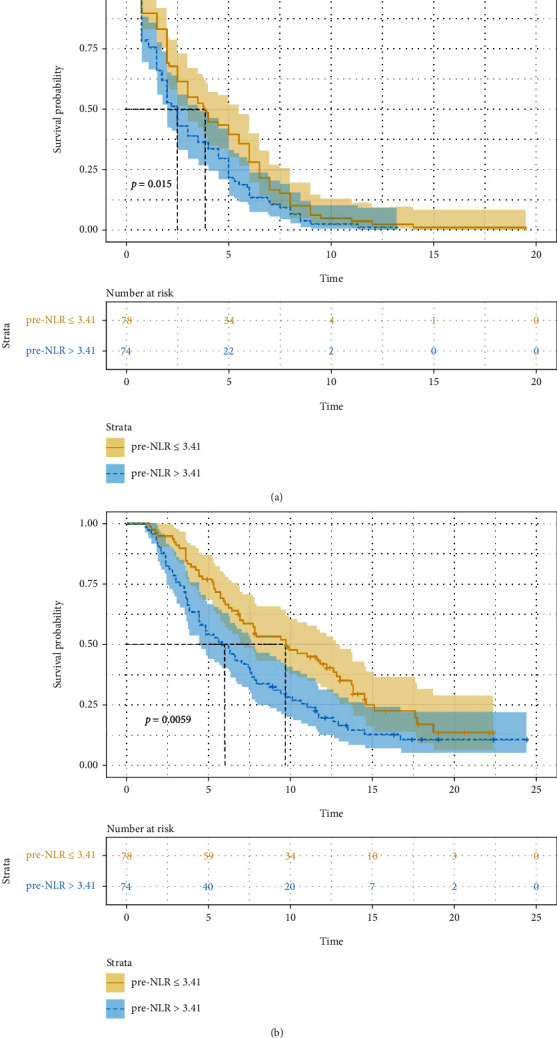
Association of pre-NLR (≤ 3.41 versus 3.41) with progress free survival ((a) *P* = 0.015) and overall survival ((b) *P* = 0.006).

**Figure 3:**
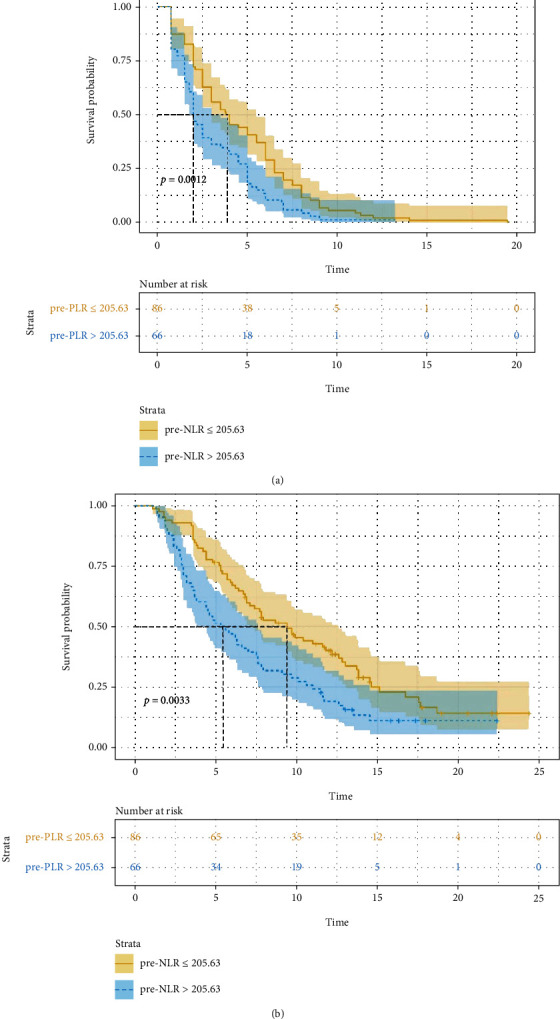
Association of pre-PLR (≤ 205.63 versus 205.63) with progress free ((a) *P* = 0.001) survival and overall survival ((b) *P* = 0.003).

**Figure 4:**
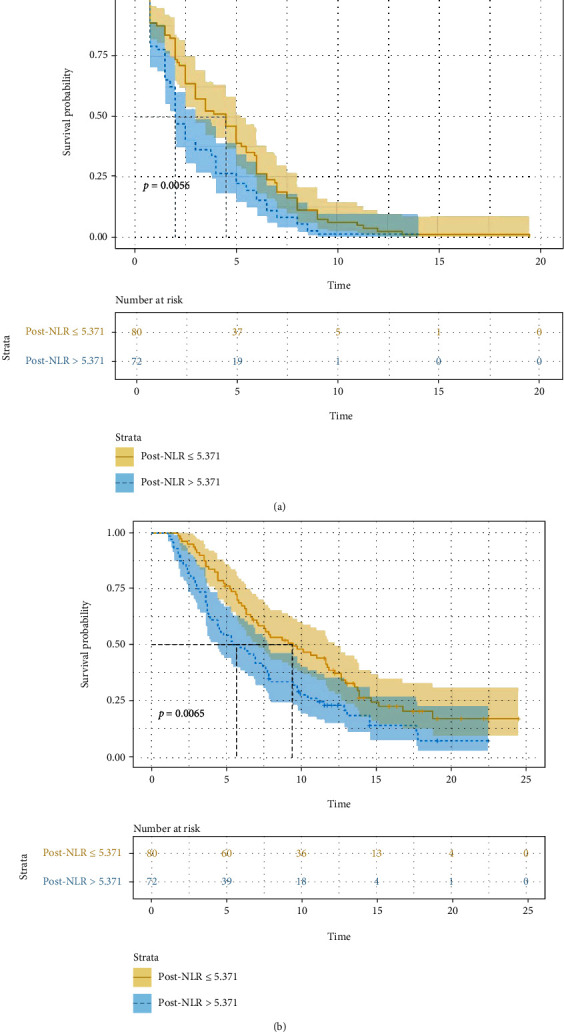
Association of post-NLR (unelevated versus elevated) with progress free survival and overall survival ((a) *P* = 0.006 and (b) *P* = 0.007).

Univariate analysis revealed that pre − NLR > 3.41, pre − PLR > 205.63, and elevated post-NLR were significant risk factors for a poor PFS or OS (Table 4). Among patients with pre − NLR > 3.41, mPFS and mOS were significantly shorter than in the other patients (2.5 months vs. 3.8 months, HR: 1.454, 95% CI: 1.054-2.008, *P* = 0.015.  Figure 2(a)) (5.8 months vs. 9.7 months, HR: 1.642, 95% CI: 1.147-2.350, *P* = 0.006.  Figure 2(b)). In patients with pre − PLR > 205.63, mPFS and mOS were significantly shorter than in patients with lower values (2.0 months vs. 3.8 months, HR: 1.654, 95% CI: 1.192-2.296, *P* = 0.001.  Figure 3(a)) (5.3 months vs. 9.4 months, HR: 1.698, 95% CI: 1.185-2.433, *P* = 0.003.  Figure 3(b)). For elevated post-NLR, mPFS and mOS were significantly shorter as well than in their nonelevated counterparts (2.0 months vs. 4.5 months, HR: 1.531, 95% CI: 1.108-2.115, *P* = 0.006.  Figure 4(a)) (5.4 months vs. 9.4 months, HR: 1.632, 95% CI: 1.140-2.337, *P* = 0.007.  Figure 4(b)). pre − PLR > 205.63 and elevated post-NLR was independent risk factor for poor PFS and OS as per multivariate analysis (Table 5).

### 3.5. Establishment of Prediction Model

**Figure 5:**
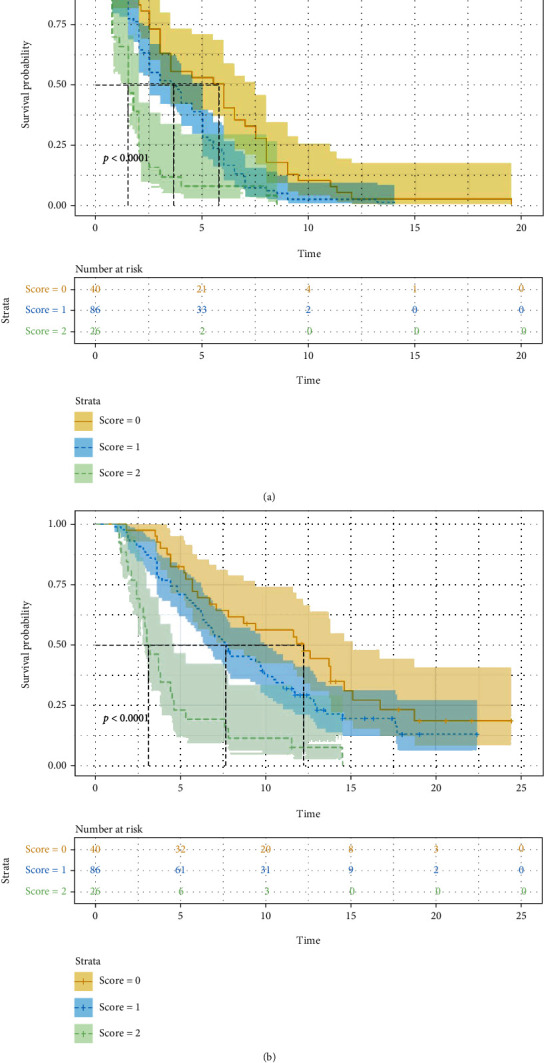
Progress free survival (PFS) and overall survival (OS) according to risk scoring ((a) *P* < 0.001 and (b) *P* < 0.001) based on pre-PLR 205.63 (+1) and elevated post-NLR (+1).

**Figure 6:**
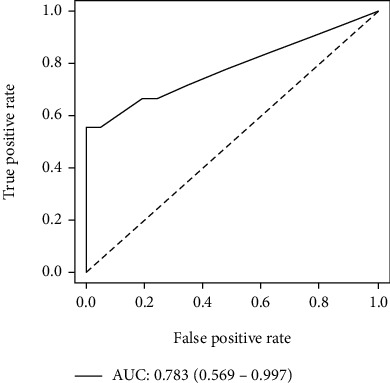
The ROC curve for the prediction model of risk scoring.

According to the results of multivariate analysis, the prediction model was established. For each risk factor, the patient's risk score increased by 1. Based on this, the patient's score can range from 0 (extremely favorable) to 2 (extremely unfavorable). Differences in scores manifested different treatment responses and prognosis (Table 6). Compared to patients with score of 1 or 2, the DCR of patients with a 0 score was statistically significantly higher and the PFS and OS longer. No statistically notable findings were made in relation to ORR; however, it showed an upward trend in patients with a score of 0 (*P* = 0.078). When PFS was evaluated according to the scoring system, the risk of death in patients with at least one risk factor was 1.85 times higher than that in patients without any risk factors (HR: 1.845, 95% CI: 1.270-2.680, P = 0.001), and the risk in patients with two risk factors was 3.21 times higher (HR: 3.205, 95% CI: 1.864-5.511, P < 0.001). When OS was assessed in a similar manner, patients with at least one risk factor were 1.77 time more at risk of death compared with patients with no risk factor (HR: 1.766, 95% CI: 1.153-2.706, *P* = 0.009). Patients with both risk factors had about 4.52 higher risk of death than those with no risk factor (HR: 4.516, 95% CI: 2.502-8.152, *P* < 0.001). There were differences in survival for the different scores (Figure 5). The prediction ability of this model was assessed using the 5-fold cross-validation (AUC: 0.783, 95% CI: 0.569-0.997), which showed favorable predictive efficacy (Figure 6).

## 4. Discussion

Our study showed a slightly lower treatment response and prognosis (DCR 71.1% vs. 81%, ORR 6.6% vs. 9.2%, PFS 3.0 vs. 5.4, and OS 7.4 vs 9.6.) when contrasted with the ALTER-0303 study. We speculate that the reasons for this discrepancy may be attributable to the differences in the study populations of the two. Patients in our study grossly speaking were with more advanced disease and worse ECOG scores, while using anlotinib as a relatively later line of therapy. The inclusion criteria of ALTER-0303 included stage IIIB patients and those using anlotinib as second-line therapy. Despite anlotinib exhibiting a small but indisputable benefit to survival in advanced NSCLC patients as third or further-line treatment, the absence of predictive factors necessitated a careful risk-benefit analysis before use in clinical practice. So, it is very important to identify appropriate patients according to the prognostic indexes so as to obtain an individualized treatment.

Neutrophils secrete cytokines and chemokines, which promote tumor progression and induce resistance to cytotoxic drugs [25]. Furthermore, platelets are an important source of cytokines, such as vascular endothelial growth factor (VEGF), which contributes to angiogenesis and cell invasion. Lymphocytes can secrete a variety of cytokines to prevent the occurrence of tumor from the immune region and regulate the process of immune surveillance. A decreased lymphocyte count reduces the antitumor response [26, 27]. Resulting from three factors stated above, it is not surprising that NLR and PLR are commonly thought to predict the prognosis of different treatments' regimens in various solid tumors including lung cancer [11, 13, 28, 29]. An article primarily focuses on the value of PLR and NLR in predicting the prognosis of different treatments in esophageal cancer was released by our team recently [30]. Although NLR and PLR have been proven to be of huge significance for predicting prognosis patients with NSCLC in respect to various treatments such as surgery, chemoradiotherapy, or immunotherapy but without a systematically evaluated model involving a combination of NLR and PLR. In this retrospective study, we selected NLR and PLR to evaluate the treatment response and prognosis in advanced NSCLC patients receiving anlotinib as a third or further-line treatment. Anlotinib's mode of action is similar to that of another antiangiogenic agents in colorectal cancer and liver cancer. Clinical inflammatory biomarkers could be used as predictors of anlotinib with varying degrees [21–24]. Our study shows that DCR is related to pre-NLR, pre-PLR, and post-NLR but bares no relationship with post-PLR. None of the factors had effect or impact on ORR. Univariate logistic regression analysis comparatively showed a long PFS and OS among patients with low values of pre-NLR, pre-PLR, or post-NLR. Multivariate logistic regression analysis on the other hand singled out only pre-PLR and post-NLR as closely associated with survival. The insignificance of pre-NLR may be in part due to the various targets of anlotinib or some unknown factor. We further demonstrated that the status of post-NLR has an independent prognostic effect as was found by Jing Wang et al. Thus, we established a risk score model to predict the prognosis including two related factors based on the results of the multivariate analysis (0 score: low pre-PLR and low post-NLR, 1 score: low pre-PLR or low post-NLR, and 2 score: high pre-PLR and high post-NLR). Our analysis showed that patients with score of 0 seemed to significantly benefit the most from the use of anlotinib in regards to PFS and OS. Such patients generally presented with an upward ORR trend; however, this was not statistically relevant. This may be a result of not only the poor ORR of anlotinib therapy (6.6%) but also the relatively small number of patients enrolled in our study. According to this model, we could not only be able to distinguish patients likely to achieve DCR but also potentially screen for those most likely to obtain ORR.

In conducting the study, we first systematically evaluated the predictive value of NLR, PLR, and their variations on third- or further-line anlotinib therapy for advanced NSCLC patients. Then, subsequently proved pre-PLR's value in predicting prognosis of patients on the regimen. The risk model detailed herein could be appropriate and useful in predicting treatment effect of anlotinib therapy as well as prognosis. It is important to note that our study was limited by it being a single center study with a relatively small sample size, hence with a minimal PR. Both factors introducing varying levels of bias. Since all patients were treated with anlotinib as at least third-line, it may have had some unknown impact on either subsequent therapy or on prognosis. Besides, a data-driven analysis often provides optimistic results. Consequently, validation analysis with independent data sets would be required in the future.

## 5. Conclusions

Pre-PLR and post-NLR are generally inexpensive and easy-to-use hematological markers that have some relationship with treatment response and, per our study, were independent prognostic indicators for patients with advanced NSCLC receiving anlotinib as a third or further-line treatment. Notably, a low pre-PLR prior to anlotinib therapy could help in stratification patients on anlotinib therapy as it produced a considerable therapeutic benefit. At the same time, the patients whose risk value score was 0, as calculated from a combination of a low pre-PLR prior anlotinib therapy and the subsequent post-NLR, were shown to have superior effectiveness and better survival.

## Figures and Tables

**Table 1:** Patients' basic characteristics.

Characteristics	Patients (%)
Age (years)	
Median	63 years
Range	32-84
<65	91 (59.9%)
≥65	61 (40.1%)
Gender	
Male	110 (72.4%)
Female	42 (27.6%)
Performance status (ECOG)	
0-1	110 (72.4%)
2-3	42 (27.6%)
Pathology	
Adenocarcinoma	69 (45.4%)
Squamous carcinoma and others	83 (54.6%)
Driver gene EGFR/ALK/c-met	
Mutant type	32 (21.1%)
Wild type	120 (78.9%)
Metastasis sites	
≤3	82 (53.9%)
>3	70 (46.1%)
History of tumor surgery	
Yes	66 (43.4%)
No	86 (56.6%)
Number of previous treatment lines	
3	89 (58.6%)
>3	63 (41.4%)

**Table 2:** Associations of NLR, PLR, and their variations with clinicopathological characteristics.

Characteristics	Pre-NLR			Pre-PLR			Post-NLR variation			Post-PLR variation		
	≤3.41	> 3.41	*P* value	≤205.63	>205.63	*P* value	Decrease	Rise	*P* value	Decrease	Rise	*P* value
Age (years)			0.221			0.244			0.714			0.468
<65	43 (28.3%)	48 (31.6%)		48 (31.6%)	43 (28.3%)		49 (32.2%)	42 (27.6%)		59 (38.8%)	32 (21.1%)	
≥65	35 (23.0%)	26 (17.1%)		38 (25.0%)	21 (15.1%)		31 (20.4%)	30 (19.8%)		36 (23.7%)	25 (16.4%)	
Gender			0.871			0.121			0.293			0.512
Male	56 (36.8%)	54 (35.5%)		58 (38.2%)	52 (34.2%)		55 (36.2%)	55 (36.2%)		67 (44.1%)	43 (28.3%)	
Female	22 (14.5%)	20 (13.2%)		28 (18.4%)	14 (9.2%)		25 (16.4%)	17 (11.2%)		28 (18.4%)	14 (9.2%)	
Pathology			0.646			0.519			0.918			0.966
Adenocarcinoma	34 (22.4%)	35 (23.0%)		41 (27.0%)	28 (18.4%)		36 (23.7%)	33 (21.7%)		43 (28.3%)	26 (17.1%)	
Squamous carcinoma and others	44 (28.9%)	39 (25.7%)		45 (29.6%)	38 (25.0%)		44 (28.9%)	39 (25.7%)		52 (34.2%)	31 (20.4%)	
Performance status			0.573			0.519			0.745			0.399
0-1	58 (38.2%)	52 (34.2%)		64 (42.1%)	46 (30.3%)		57 (37.5%)	53 (34.9%)		71 (46.7%)	39 (25.7%)	
2-3	20 (13.2%)	22 (14.4%)		22 (14.5%)	20 (13.1%)		23 (15.1%)	19 (12.5%)		24 (15.8%)	18 (11.8%)	
Driver gene EGFR/ALK/c-met			0.530			0.118			0.950			0.100
Mutant type	18 (11.8%)	14 (9.2%)		22 (14.5%)	10 (6.6%)		17 (11.2%)	15 (9.9%)		24 (15.8%)	8 (5.3%)	
Wild type	60 (39.5%)	60 (39.5%)		64 (42.1%)	56 (36.8%)		63 (41.4%)	57 (37.5%)		71 (46.7%)	49 (32.2%)	
Number of metastases			0.532			0.598			0.706			0.556
≤3	44 (28.9%)	38 (25.0%)		48 (31.6%)	34 (22.4%)		42 (27.6%)	40 (26.3%)		53 (34.9%)	29 (19.1%)	
>3	34 (22.4%)	36 (23.7%)		38 (25.0%)	32 (21.1%)		38 (25.0%)	32 (21.1%)		42 (27.6%)	28 (18.4%)	
History of tumor surgery			0.711			0.380			0.679			0.108
No	43 (28.3%)	43 (28.3%)		46 (30.3%)	40 (26.3%)		44 (28.9%)	42 (27.6%)		49 (32.2%)	37 (24.3%)	
Yes	35 (23.0%)	31 (20.4%)		40 (26.3%)	26 (17.1%)		36 (23.7%)	30 (19.7%)		46 (30.3%)	20 (13.2%)	
Number of previous treatment lines			0.582			0.380			0.089			0.251
3	44 (28.9%)	45 (29.6%)		53 (34.9%)	36 (23.7%)		52 (34.2%)	37 (24.3%)		59 (38.8%)	30 (19.7%)	
>3	34 (22.4%)	29 (19.1%)		33 (21.7%)	30 (19.7%)		28 (18.4%)	35 (23.1%)		36 (23.7%)	27 (17.8%)	

All the *P* values are obtained with chi-square.

**Table 3:** Associations of NLR, PLR, and their variations with treatment response.

Treatment response	Pre-NLR			Pre-PLR			Post-NLR variation			Post-PLR variation		
	≤ 3.41	> 3.41	*P* value	≤ 205.63	>205.63	*P* value	Decrease	Rise	*P* value	Decrease	Rise	*P* value
PR	7 (4.6%)	3 (2.0%)	0.019^∗∗^	7 (4.6%)	3 (2.0%)	0.016^∗∗^	6 (3.9%)	4 (2.6%)	0.037^∗∗^	5 (3.3%)	5 (3.3%)	0.642^∗∗^
SD	56 (36.8%)	42 (27.6%)		62 (40.8%)	36 (23.7%)		58 (38.2%)	40 (26.3%)		61 (40.1%)	37 (24.3%)	
PD	15 (9.9%)	29 (19.1%)		17 (11.2%)	27 (17.8%)		16 (10.5%)	28 (18.4%)		29 (19.1%)	15 (9.9%)	
ORR	4.6%	2.0%	0.329^∗∗^	4.6%	2.0%	0.515^∗∗^	3.9%	2.6%	0.749^∗^	3.3%	3.3%	0.398^∗^
DCR	41.4%	29.6%	0.007^∗^	45.4%	25.7%	0.004^∗^	42.1%	28.9%	0.010^∗∗^	43.4%	27.6%	0.579^∗∗^

^∗^analyzed with chi-square test. ^∗∗^analyzed with Fisher.

**Table 4:** Univariate analysis of factors associated with progression free survival and overall survival.

Prognostic factors	Patients	Progression free survival			Overall survival		
		HR	95% CI	*P* value	HR	95% CI	*P* value
Age (years)							
<65	91	1			1		
≥65	61	0.936	0.673-1.300	0.693	0.914	0.634-1.319	0.632
Gender							
Female	42	1			1		
Male	110	1.091	0.762-1.560	0.635	1.373	0.907-2.078	0.134
Pathology							
Squamous carcinoma and others	83	1			1		
Adenocarcinoma	69	0.926	0.669-1.281	0.641	1.069	0.746-1.531	0.718
Performance status							
0-1	110	1			1		
2-3	42	1.033	0.721-1.480	0.860	1.461	0.986-2.166	0.059
Driver gene EGFR/ALK/c-met							
Wild type	120	1			1		
Mutant type	32	0.830	0.558-1.235	0.358	0.834	0.536-1.300	0.423
Number of metastases							
≤3	82	1			1		
>3	70	1.044	0.756-1.441	0.796	1.091	0.762-1.561	0.634
History of tumor surgery							
No	86	1			1		
Yes	66	0.813	0.586-1.128	0.214	0.734	0.509-1.059	0.099
Number of previous treatment lines							
3	89	1			1		
>3	63	1.147	0.828-1.588	0.409	1.020	0.710-1.466	0.913
Pre-NLR							
≤3.41	78	1			1		
>3.41	74	1.454	1.054-2.008	0.015	1.642	1.147-2.350	0.006
Pre-PLR							
≤205.63	86	1			1		
>205.63	66	1.654	1.192-2.296	0.001	1.698	1.185-2.433	0.003
Post-NLR variation							
Decrease	80	1			1		
Rise	72	1.531	1.108-2.115	0.006	1.632	1.140-2.337	0.007
Post-PLR variation							
Decrease	95	1			1		
Rise	57	0.982	0.706-1.366	0.915	1.125	0.778-1.625	0.532

**Table 5:** Multivariable analysis of factors associated with progression free survival and overall survival.

Prognostic factors	Progression free survival			Overall survival		
	HR	95% CI	*P* value	HR	95% CI	*P* value
Pre-NLR (≤ 3.41 vs. >3.41)	1.200	0.760-1.895	0.433	1.408	0.862-2.299	0.171
Pre-PLR (≤ 205.63 vs. >205.63)	1.642	1.036-2.603	0.035	1.655	1.004-2.729	0.048
Post-NLR variation (decrease vs. rise)	1.772	1.268-2.477	0.001	2.047	1.404-2.984	0.001

**Table 6:** Treatment response and prognosis for different patient scores stratified according to independent prognostic factors (pre-PLR level and post-NLR variation).

Treatment response and prognosis	Score			*P* value
	0	1	2	
DCR	87.5%	73.3%	38.5%	0.001^∗^
ORR	12.5%	3.5%	7.7%	0.175^∗∗^
mPFS (months)	5.5	3.5	1.5	0.001
mOS (months)	12.2	7.6	3	0.001

0: patients negative for both risk factors (pre-PLR level and post-NLR variation). 1: patients with 1 of the above mentioned prognostic factors. 2: patients with both prognostic factors. ^∗^analyzed with chi-square test. ^∗∗^analyzed with Fisher.

## Data Availability

The data used to support the findings of this study are available from the corresponding author upon request.
